# coBRIM: a phase 3, double-blind, placebo-controlled study of vemurafenib versus vemurafenib + cobimetinib in previously untreated *BRAF*^V600^ mutation–positive patients with unresectable locally advanced or metastatic melanoma (NCT01689519)

**DOI:** 10.1186/1479-5876-13-S1-O4

**Published:** 2015-01-15

**Authors:** Paolo A Ascierto, Grant A McArthur, Brigitte Dréno, James Larkin, Gabriella Liszkay, Michele Maio, Mario Mandala, Lev Demidov, Daniil Stroyakovskiy, Luc Thomas, Luis de la Cruz-Merino, Victoria Atkinson, Caroline Dutriaux, Claus Garbe, Ilsung Chang, Stephen P Hack, Antoni Ribas

**Affiliations:** 1 Istituto Nazionale Tumori Fondazione G. Pascale, Naples, Italy; 2 Peter MacCallum Cancer Centre, Melbourne, Victoria, Australia; 3 Hôtel Dieu Place Alexis Ricordeau, Nantes, France; 4 The Royal Marsden Hospital, London, UK; 5 National Institute of Oncology, Budapest, Hungary; 6 Azienda Ospedaliera Universitaria Senese, Siena, Italy; 7 Papa Giovanni XXIII Hospital, Bergamo, Italy; 8 N.N. Blokhin Russian Cancer Research Center, Moscow, Russia; 9 Moscow City Oncology Hospital 62, Krasnogorsk, Russia; 10 Centre Hospitalier Lyon Sud, Pierre-Bénite, France; 11 Hospital Universitario Virgen Macarena, Seville, Spain; 12 Princess Alexandra Hospital, Woolloongabba, QLD, Australia; 13 Hopital Saint André, Bordeaux, France; 14 University of Tübingen, Tübingen, Germany; 15 Genentech, Inc, South San Francisco, CA, USA; 16 Jonsson Comprehensive Cancer Center at the University of California, Los Angeles, Los Angeles, CA, USA

## Background

Combined inhibition of BRAF and MEK is hypothesized to improve clinical outcomes by preventing or delaying onset of resistance observed with BRAF inhibitors alone. This randomized phase 3 study evaluated the combination of the BRAF inhibitor vemurafenib and the MEK inhibitor cobimetinib.

## Materials and methods

495 patients were randomly assigned to receive vemurafenib + cobimetinib (60 mg QD, 21 days on/7 days off) or vemurafenib (960 mg BID) + placebo. Eligibility included treatment-naive *BRAF*^V600^ mutation–positive patients with unresectable locally advanced or metastatic melanoma and adequate performance status and organ function. The primary end point was investigator-assessed progression-free survival (PFS). Safety monitoring included serial cardiac and ophthalmic evaluation and measurement of creatine phosphokinase.

## Results

Median PFS was 9.9 months with the combination compared with 6.2 months with the control (HR, 0.51; 95% CI, 0.39-0.68; *P*<0.0001). Objective response rate (ORR) was 68% in the combination and 45% in the control arm (*P*<0.0001), including complete response in 10% in the combination and 4% of patients in the control group. Subgroup analyses of PFS based on key demographic and tumor characteristics were consistent with PFS in the intent-to-treat population, including those with normal or elevated baseline lactate dehydrogenase (LDH). PFS assessed by independent review was comparable with investigator-assessed PFS. Interim overall survival (OS) data showed an HR of 0.65 (95% CI, 0.42-1.00) but did not cross the prespecified stopping boundary. Compared with vemurafenib alone, the combination was associated with a higher incidence of grade 3 or 4 adverse events (65% vs 59%), with no difference in the rate of adverse events leading to study drug discontinuation (13% vs 12%). Most grade ≥3 events occurred in the first 28 days and resolved quickly. Known MEK inhibitor–related toxicities such as diarrhea, serous retinopathy, elevated creatine phosphokinase, and increased liver transaminase levels were more commonly observed with the combination. The majority was grade 1 or 2, occurred between 1 and 4 months in the treatment course, and resolved quickly. The occurrence of secondary cutaneous neoplasms decreased with the combination (4% vs 18%). Photosensitivity was more common in patients treated with the combination (all grades 32% vs 18%).

## Conclusion

Cobimetinib + vemurafenib significantly improved PFS and response rate among patients with *BRAF*^V600^-mutant metastatic melanoma, with promising preliminary OS analysis. Most combination-related toxicities are mild or moderate, occur early in treatment, and are manageable by dose modification and supportive care; treatment discontinuation is uncommon.

## Clinical trial registration number

NCT01689519

